# Pharmacological Evaluation of a Traditional Thai Polyherbal Formula for Alzheimer’s Disease: Evidence from In Vitro and In Silico Studies

**DOI:** 10.3390/ijms26136287

**Published:** 2025-06-29

**Authors:** Pornthip Waiwut, Pitchayakarn Takomthong, Rutchayaporn Anorach, Nattareeyada Lomaboot, Supawadee Daodee, Yaowared Chulikhit, Orawan Monthakantirat, Charinya Khamphukdee, Chantana Boonyarat

**Affiliations:** 1 Faculty of Pharmaceutical Sciences, Ubon Ratchathani University, Ubon Ratchathani 34190, Thailand; pwaiwut79@yahoo.com; 2 Faculty of Pharmaceutical Sciences, Khon Kaen University, Khon Kaen 40002, Thailand; ppitcha.t@gmail.com (P.T.); rutano@kku.ac.th (R.A.); aumlom@kku.ac.th (N.L.); csupawad@kku.ac.th (S.D.); yaosum@kku.ac.th (Y.C.); oramon@kku.ac.th (O.M.); charkh@kku.ac.th (C.K.)

**Keywords:** *Oroxylum indicum*, *Zingiber officinale*, *Boesenbergia rotunda*, network pharmacology, cholinesterase, amyloid-beta aggregation, neuroprotection, oxidative stress

## Abstract

Alzheimer’s disease (AD) is a complex neurodegenerative disorder characterized by multifactorial pathogenesis, including oxidative stress, cholinergic dysfunction, β-amyloid (Aβ) aggregation, and neuroinflammation. In this study, we investigated the neuroprotective potential of the Pheka capsule (PC) formula, a traditional Thai polyherbal medicine comprising *Oroxylum indicum* (OI), *Zingiber officinale* (ZO), and *Boesenbergia rotunda* (BR). Phytochemical analysis by HPLC confirmed the presence of key bioactive compounds including baicalein, baicalin, oroxylin A, 6-gingerol, 6-shogaol, pinocembrin, and pinostrobin. The PC formula exhibited strong antioxidant activity, highly selective butyrylcholinesterase (BChE) inhibition with a selectivity index (SI) of BChE > 20, suppression of Aβ aggregation, and protection against H_2_O_2_-induced neuronal damage in vitro. Network pharmacology analysis identified multiple AD-relevant targets and pathways, including APP, GSK3B, CASP3, GAPDH, PTGS2, and PPARG, implicating the PC formula in modulating oxidative stress, apoptosis, and inflammation. Notably, OI emerged as the primary contributor to the formula’s multitargeted actions. These findings support the therapeutic potential of the PC formula as a multitarget agent for AD, aligning with the growing interest in polypharmacological strategies for complex neurodegenerative diseases. Further in vivo and clinical studies are warranted to confirm its efficacy and safety.

## 1. Introduction

Alzheimer’s disease (AD) is a chronic, progressive neurodegenerative disorder and the most prevalent cause of dementia, currently affecting over 55 million individuals worldwide [1]. It is clinically characterized by a gradual deterioration in memory, cognition, and executive function, often accompanied by neuropsychiatric symptoms such as depression, anxiety, and apathy, which contribute to a significant decline in quality of life and an increasing burden on healthcare systems [1,2]. With global aging trends, the incidence of AD is projected to rise dramatically, underscoring the urgent need for innovative and effective disease-modifying therapies.

AD pathogenesis is multifactorial and complex, involving a cascade of interrelated molecular events including extracellular deposition of β-amyloid (Aβ) plaques, intracellular accumulation of hyperphosphorylated tau protein in neurofibrillary tangles, synaptic and neuronal degeneration, mitochondrial dysfunction, oxidative stress, and chronic neuroinflammation [3]. Despite intensive research and development, current pharmacotherapies—namely acetylcholinesterase inhibitors and NMDA receptor antagonists—provide only limited symptomatic relief and do not alter the underlying disease course [4]. Given the heterogeneous and interconnected nature of AD pathology, there is growing interest in multi-target therapeutic strategies capable of modulating multiple disease pathways simultaneously [5].

Natural products derived from medicinal plants have garnered increasing attention as potential sources of neuroprotective agents [6,7,8]. These bioactive compounds—such as polyphenols, flavonoids, terpenoids, and alkaloids—are known to exert antioxidant, anti-inflammatory, anti-amyloidogenic, and cholinesterase inhibitory activities at the molecular level [9,10,11]. These multi-functional properties position them as promising candidates for the development of safer, more holistic therapeutic approaches to combat neurodegeneration.

Traditional Thai Medicine (TTM), a holistic and integrative healthcare system with centuries of empirical use, provides a rich repository of plant-based remedies [12,13]. TTM incorporates herbal medicine, diet, massage, and spiritual practices and emphasizes prevention and systemic balance [12,14]. Central to TTM are polyherbal formulations—combinations of medicinal plants designed to act synergistically across multiple biological targets while minimizing adverse effects [15]. Recent pharmacological investigations have begun to validate the neuroprotective efficacy of TTM-derived herbs and formulations, many of which contain bioactive constituents relevant to AD mechanisms, including neuroinflammation, oxidative stress, cholinergic dysfunction, and Aβ pathology [6,8,16,17].

A noteworthy example of a traditional Thai polyherbal preparation is the Pheka capsule (PC), which has long been used by local healers in Thailand as a multi-herb folk remedy for general health promotion [18,19]. This traditional formula has since been standardized into capsule form for clinical application at Chao Phya Abhaibhubejhr Hospital in Thailand, where it is prescribed by Thai traditional medicine practitioners to support overall well-being and enhance blood circulation. A prior clinical trial established the safety and efficacy of PC in reducing LDL cholesterol in patients with hypercholesterolemia, demonstrating no adverse impact on renal, hepatic, or muscular enzyme levels [19]. The PC formula consists of *Oroxylum indicum* (OI), *Zingiber officinale* (ZO), and *Boesenbergia rotunda* (BR), mixed in a ratio of 280:80:40 (dry weight). These plants are widely used both as functional foods and traditional remedies across Southeast Asia, and it is particularly interesting that each component has reported biological activity relevant to AD. Molecular characterization reveals that OI is rich in flavonoids such as baicalein, baicalin, chrysin, and oroxylin, which exhibit strong antioxidant and anti-inflammatory activities and have shown cognitive-enhancing effects in preclinical models [8,20,21]. ZO demonstrates cholinesterase inhibitory, anti-oxidative, and neuroprotective properties, with active compounds such as 6-gingerol contributing to its pharmacological profile [20,22,23,24,25,26]. BR contains flavonoids and essential oils that exhibit neuroprotective, anti-inflammatory, and antioxidant actions relevant to neurodegenerative disorders [27,28]. These characteristics suggest that the PC formula may possess multifaceted mechanisms relevant to AD prevention and therapy.

The present study aimed to systematically evaluate the neuroprotective potential of the PC formula against key pathological processes of AD. To achieve this, we adopted a two-pronged strategy. First, we assessed its multitarget in vitro effects, including antioxidant activity, inhibition of Aβ aggregation, acetylcholinesterase inhibition, and protection against oxidative stress-induced neuronal damage. Second, we applied a network pharmacology approach to identify the target genes, and molecular pathways involved in its therapeutic effects. This integrative strategy provides a comprehensive understanding of the formula’s action at a systems biology level, with a focus on the molecular mechanisms relevant to AD.

By combining traditional medicinal knowledge with modern molecular and computational methodologies, this study seeks to scientifically validate the therapeutic potential of a traditional polyherbal remedy for AD. Our findings contribute to the growing body of evidence supporting natural products as viable sources of multi-target therapeutics for neurodegenerative diseases and highlight the importance of ethnopharmacological approaches in modern drug discovery.

## 2. Results

### 2.1. In Vitro Experimental Validation Results

#### 2.1.1. Preparation of the PC Formula and Each Herbal Plant Extract

The PC formula consists of *Oroxylum indicum* (OI), *Zingiber officinale* (ZO), and *Boesenbergia rotunda* (BR) in a ratio of 280:80:40, which followed the Thai traditional formula. A dried powder mixture (200 g) and each herbal plant (50 g) were macerated in 95% ethanol at room temperature for 7 d and then filtered. The extracts were concentrated under reduced pressure and freeze-dried, yielding percentages of 11.78, 12.82, 6.46, and 11.86 for the PC formula, OI, ZO, and BR, respectively. All crude extracts were kept at −20 °C during the experiment.

#### 2.1.2. Identification of PC Formula Components by High-Performance Liquid Chromatography (HPLC)

**Figure 1:**
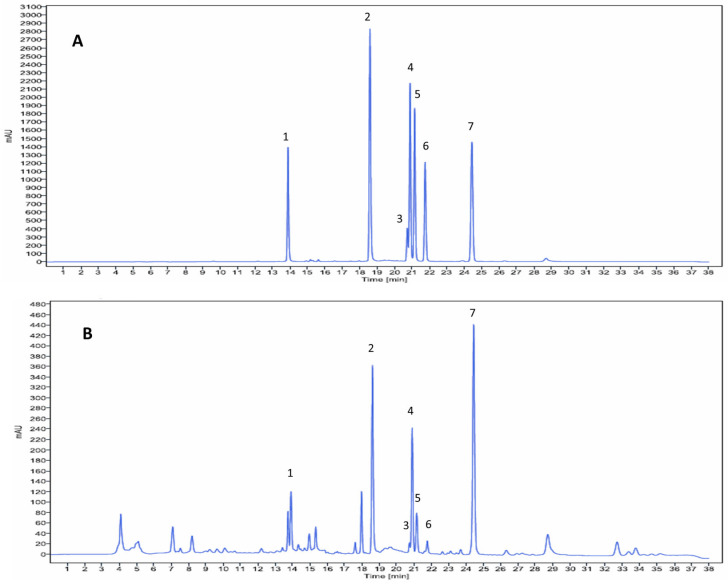
HPLC chromatogram of standard mixture (**A**) and PC formula extract (**B**) in mobile phase system of 1% formic acid (**A**) and 10% methanol in acetonitrile (**B**) at a flow rate of 0.5 mL/min. The gradient program ran from 70% A to 5% A. Peak 1 = baicalin (1), peak 2 = baicalein (2), peak 3 = 6-gingerol (3), peak 4 = pinocembrin (4), peak 5 = chrysin (5), peak 6 = oroxylin A (6), peak 7 = pinostrobin (7).

The PC formula, comprising OI, ZO, and BR, is known to possess a diverse array of chemical constituents. Based on a comprehensive review of the literature and databases, we identified several key active phytochemicals reported in these herbal plants, including 6-gingerol, pinocembrin, pinostrobin, oroxylin A, chrysin, baicalein, and baicalin [25,29,30,31,32,33]. Our HPLC analysis of the PC formula extract confirmed the presence of these major compounds: baicalin (1), baicalein (2), 6-gingerol (3), pinocembrin (4), chrysin (5), oroxylin A (6), and pinostrobin (7), as shown in Figure 1.

#### 2.1.3. Determination of Total Phenolic (TPC) and Flavonoid Contents (TFC)

TPC and TFC were determined from their calibration curves (r^2^ = 0.9991 and r^2^ = 0.9996, respectively), and the results of PC formula and each extract were shown in Table 1. ZO exhibited the highest TPC at 205.46 ± 7.65 mg GAE/g extract, followed by BR and OI, with TPC values of 83.29 ± 5.96 and 62.58 ± 1.22 mg GAE/g extract, respectively. Additionally, BR also demonstrated the highest TFC at 131.81 ± 3.22 mg QE/g crude extract, compared to ZO and OI, which had TFC values of 110.48 ± 4.06 and 14.83 ± 0.65 mg QE/g crude extract, respectively. The TPC and TFC of PC formula were 73.65 ± 1.39 mg GAE/g extract and 31.86 ± 1.13 mg QE/g crude extract, respectively.

#### 2.1.4. Antioxidant Effect by DPPH and ABTS Assays

**Figure 2:**
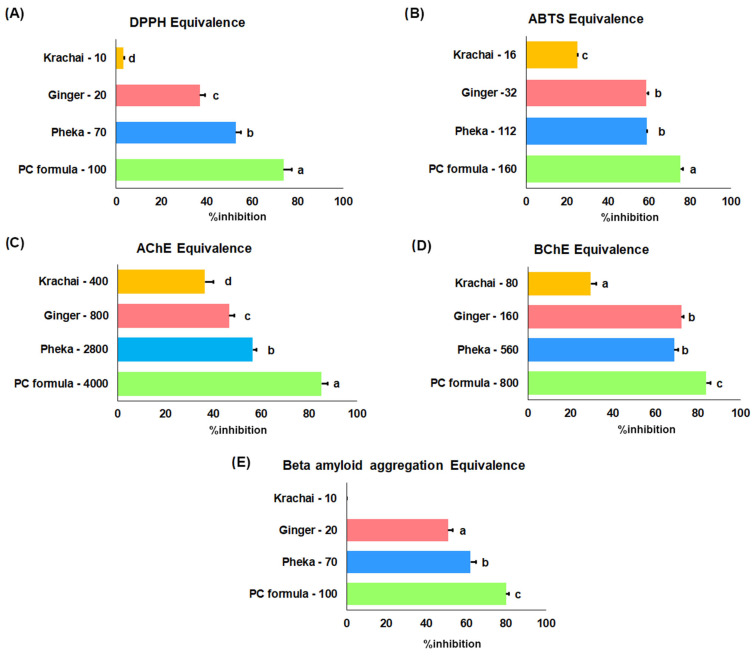
Equivalence assay of PC formula and its plant constituents. The radical scavenging activity (DPPH assay in (**A**), ABTS assay in (**B**)), cholinesterase inhibitory effects (AChE in (**C**), BChE in (**D**)), and inhibition of Aβ aggregation (**E**) were evaluated for the complete PC formula and its individual components at equivalent concentrations. Data are presented as means ± standard deviation (*n* = 3). Statistical significance (*p* < 0.05) between groups is indicated by different letters based on the Kruskal–Wallis test followed by pairwise Mann–Whitney U tests.

The results, summarized in Table 2, provide the inhibition concentration (IC_50_; µg/mL) values for each sample, indicating their efficacy in scavenging free radicals. ZO was found to be the most effective herbal plant in both assays, with IC_50_ values of 27.99 ± 2.79 µg/mL against DPPH and 24.01 ± 1.05 µg/mL against ABTS. To investigate whether the individual herbal plants exerted antioxidant effects within the PC formula, the equivalence assays of DPPH and ABTS were performed based on the plant ratio (Figure 2A,B). The results revealed that OI is the most effective herbal plant in both the DPPH and ABTS assays, likely due to its higher concentration in the formula. However, in the ABTS assay, there was no significant difference between the efficacy of OI and ZO. This suggested that ZO has significant potential to scavenge ABTS free radicals effectively in PC formula.

#### 2.1.5. Cholinesterase Inhibition

There are two types of cholinesterase: acetylcholinesterase (AChE) and butyrylcholinesterase (BChE). Both enzymes play roles in cholinergic transmission and AD progression. In this study, the PC formula and each herbal plant were evaluated for their inhibitory effects on these enzymes, as shown in Table 3. Among the herbal extracts, ZO exhibited the most potent enzyme inhibition. However, the PC formula demonstrated the highest selectivity for BChE, with a selectivity index of 24.69. Further analysis was conducted to identify which extracts most significantly contributed to the PC formula in cholinesterase inhibition (Figure 2C,D), revealing that OI has the greatest potential to inhibit both enzymes, while ZO also shows potential specifically against BChE.

#### 2.1.6. Aβ Aggregation

Aβ is one of the AD hallmarks. The deposition of beta amyloid can lead to the formation of plaques, which disrupt cell communication and trigger inflammatory responses in the brain. This accumulation can also induce oxidative stress and neuronal toxicity, ultimately contributing to synaptic dysfunction, neuronal death, and cognitive decline in AD. Therefore, the PC formula and each herbal extract were evaluated for their ability to inhibit Aβ aggregation using the thioflavin T assay. Among the groups, the PC formula and ZO showed the highest ability to inhibit beta amyloid aggregation (Table 4). Unfortunately, BR did not exhibit this inhibitory effect. Further analysis revealed that OI is the extract with the greatest potential to contribute to the ability of PC to inhibit Aβ aggregation (Figure 2E).

#### 2.1.7. Neuroprotective Effects

**Figure 3:**
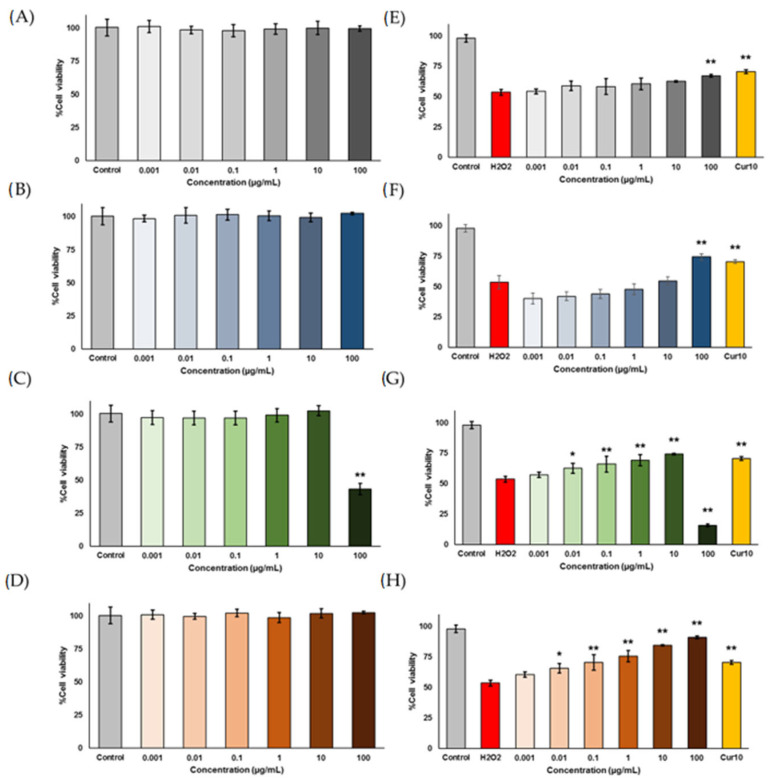
Cytotoxicity (**A**–**D**) and neuroprotective effects against H_2_O_2_-induced SH-SY5Y cell damage (**E**–**H**) of PC formula, OI, BR, and ZO. Cell viability of SH-SY5Y cells was assessed using the MTT assay. Panels (**E**–**H**) show the neuroprotective effects following pre-treatment with 400 µM H_2_O_2_ to induce oxidative damage, followed by treatment with the extracts at various concentrations. Data are means ± SD (*n* = 5). Statistical significance (* *p* < 0.05, ** *p* < 0.01) indicates a significant difference compared to the control group in the cytotoxicity assay or the H_2_O_2_-treated control in the neuroprotection assay.

First, the cytotoxicity effects of the PC formula and each individual herbal plant were investigated to assess their toxicity to SH-SY5Y neuroblastoma cells (Figure 3A–D). None of the tested samples exhibited toxicity to the cells, except for BR, which showed cytotoxic effects at a concentration of 100 µg/mL. Next, the PC formula and the individual herbal plants were further evaluated for their neuroprotective effects against hydrogen peroxide (H_2_O_2_)-induced cell damage. This evaluation aims to determine their potential in protecting neuronal cells from oxidative stress-related damage. The results demonstrated that all could protect cells from oxidative damage (Figure 3E–H).

### 2.2. Pharmacology Network Analysis

#### 2.2.1. Predicted Targets for AD

**Figure 4:**
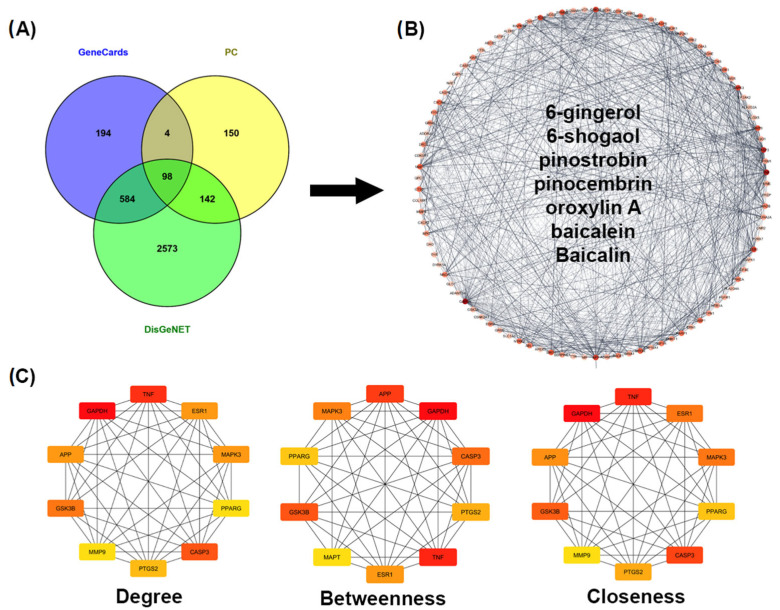
Construction and analysis of the protein–protein interaction (PPI) network related to the AD targets of the PC formula. (**A**) Venn diagram showing the overlapping target genes between the active components of the PC formula and AD-related genes. (**B**) PPI network constructed using the STRING database and visualized with Cytoscape, illustrating the interactions among the overlapping target proteins. (**C**) Identification of the top 10 hub genes ranked according to three centrality measures: degree, betweenness, and closeness, which reflect the importance of each node within the network.

Several studies have identified oroxylin A, baicalein, and baicalin as the major compounds in OI, while 6-gingerol and 6-shogaol are predominant in ZO. In BR, pinocembrin and pinostrobin are consistently used as marker compounds. Accordingly, these seven major compounds—6-gingerol, 6-shogaol, pinocembrin, pinostrobin, oroxylin A, baicalein, and baicalin—were selected for pharmacological network analysis. To predict their potential biological targets, we utilized SwissTargetPrediction, which yielded 397 unique targets after data integration and refinement. To further pinpoint targets relevant to AD, we compiled data from the GeneCards and DisGeNET databases. After eliminating duplicates, we intersected the predicted targets of the PC formula with known AD-related targets, resulting in a Venn diagram (Figure 4A). This analysis revealed 98 overlapping targets that may play a crucial role in the therapeutic potential of the PC formula against AD.

#### 2.2.2. Construction and Analysis of the Target PPI Network

We constructed a network diagram to visualize the overlap of 98 genes associated with both PC and AD targets (Figure 4B). These genes were retrieved from the STRING database and filtered using a medium confidence score of 0.4 to establish the protein–protein interaction (PPI) network. The resulting network consisted of 98 nodes and 1018 edges. Furthermore, the PPI network was analyzed using the CytoHubba plugin to identify core targets based on degree, closeness, and betweenness centrality measures. This analysis revealed key hub genes, which were identified as the primary targets of the PC formula for AD (Figure 4C).

#### 2.2.3. Gene Ontology (GO) and Kyoto Encyclopedia of Genes and Genomes (KEGG) Pathway Enrichment Analyses

**Figure 5:**
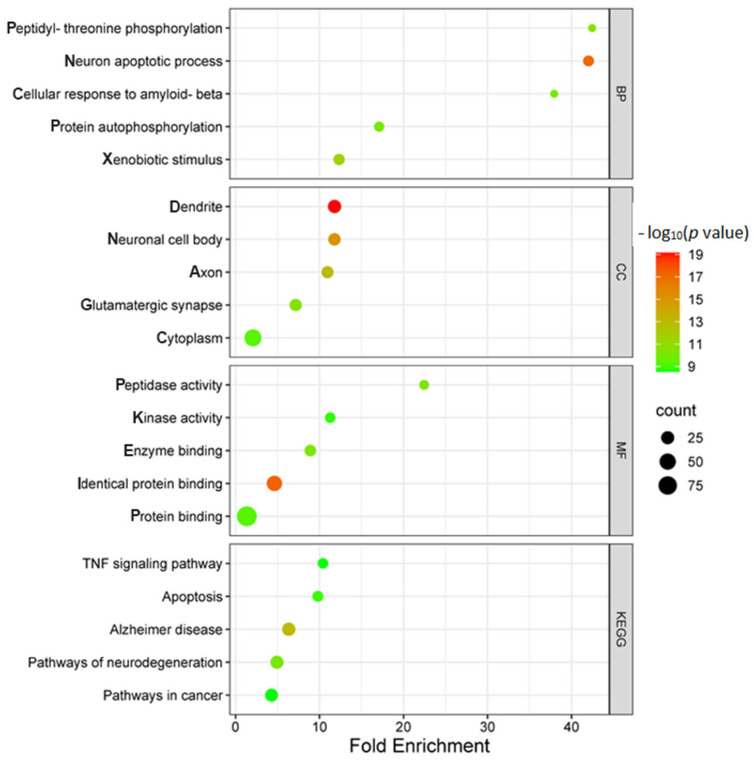
Gene ontology (GO) and KEGG pathway enrichment analysis of AD-compound-related targets represent by lollipop chart. Functional enrichment analysis was conducted using the DAVID database to identify significantly enriched biological processes (BP), cellular components (CC), molecular functions (MF), and KEGG pathways associated with the overlapping targets of AD and compounds from the PC formula.

Functional enrichment analysis was performed using DAVID to identify the most enriched biological processes (BP), cellular components (CC), and molecular functions (MF) (Figure 5). According to our findings, the effects of the PC formula on BP were primarily associated with the positive regulation of neuronal apoptosis, response to xenobiotic stimuli, peptidyl-threonine phosphorylation, cellular response to amyloid-beta, and protein autophosphorylation. For CC, the analysis revealed that most targets were enriched in the dendrites, neuronal cell bodies, axons, glutamatergic synapses, and cytoplasm. Regarding MF, the enriched functions included identical protein binding, peptidase activity, enzyme binding, protein binding, and kinase activity. KEGG pathway analysis identified several relevant pathways, including Alzheimer’s disease, neurodegeneration-associated pathways, apoptosis, the TNF signaling pathway, and cancer-related pathways. Among these, the Alzheimer’s disease pathway was highlighted as a key pathway, exhibiting the highest target enrichment and the lowest *p*-value (Supplementary File S1).

#### 2.2.4. Gene Expression Omnibus (GEO) Dataset Analysis of AD-Associated PC Targets

**Figure 6:**
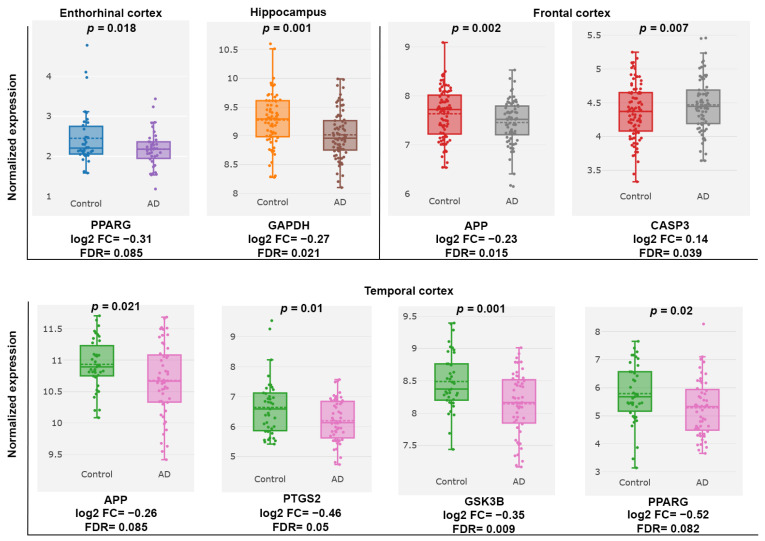
Core targets of PC formula in the control and AD groups showing cross-platform normalized expression levels in different brain regions.

Using the differential expression module of the AlzData database, we analyzed the normalized expression values of PC target genes in healthy controls and AD patients (Figure 6). Gene expression analysis across the entorhinal cortex, hippocampus, temporal cortex, and frontal cortex revealed significant dysregulation of several PC-related genes in AD patients. In the entorhinal cortex, PPARG was notably downregulated, while GAPDH showed decreased expression in the hippocampus. The temporal cortex exhibited downregulation of multiple genes, including APP, PTGS2, GSK3B, and PPARG. In contrast, the frontal cortex displayed downregulation of APP alongside an upregulation of CASP3.

## 3. Discussion

This study provides preliminary scientific support for the Pheka capsule formula, a traditional Thai polyherbal medicine, suggesting its potential as a multitarget candidate for further investigation in Alzheimer’s disease (AD). Our findings reveal that the Pheka capsule (PC) formula, composed of *Oroxylum indicum* (OI), *Zingiber officinale* (ZO), and *Boesenbergia rotunda* (BR) in a 280:80:40 ratio, exhibits a multitargeted approach relevant to AD pathology. We demonstrated its antioxidant, anticholinesterase, anti-Aβ aggregation, and neuroprotective effects against oxidative damage, suggesting a strong therapeutic potential. Furthermore, network pharmacology analysis elucidated potential mechanisms by identifying key active components, targets, and pathways through which the PC formula may exert its effects on AD.

The PC formula, comprising OI, ZO, and BR, is recognized for its diverse chemical composition. A comprehensive review of existing literature and databases allowed us to identify several key active phytochemicals reported in these constituent herbs, specifically 6-gingerol, pinocembrin, pinostrobin, oroxylin A, chrysin, baicalein, and baicalin [25,29,30,31,32,33]. Our subsequent High-Performance Liquid Chromatography (HPLC) analysis of the PC formula extract successfully confirmed the presence of these major compounds.

In parallel, we quantified the TPC and TFC of the PC formula and its individual plant components. While ZO, BR, and OI exhibited considerable TPC and TFC values individually, the PC formula demonstrated lower overall TPC (73.65 ± 1.39 mg GAE/g extract) and TFC (31.86 ± 1.13 mg QE/g crude extract). This reduction likely reflects a dilution effect resulting from the larger proportion of OI—which contains lower TPC and TFC—relative to ZO and BR.

It is well-established that flavonoids and phenolic compounds, which are significant constituents of the PC formula, possess potent capabilities to scavenge free radicals and modulate cellular signaling pathways [34]. Consistent with this, the PC formula demonstrated antioxidant activity, primarily driven by its high total phenolic and flavonoid content. Interestingly, while ZO exhibited the highest concentration of phenolic compounds, equivalence testing indicated that OI was the primary contributor to the overall antioxidant capacity of the formulation, likely due to its higher dosage within the polyherbal mixture. This observation emphasizes the crucial role of both individual compound concentration and potential synergistic interactions in the overall efficacy of polyherbal formulations.

The primary treatment for alleviating cognitive symptoms in AD is the use of AChE inhibitors [35]. These drugs prevent the breakdown of acetylcholine, thereby increasing its concentration in the brain and potentially improving cognitive function. Our results revealed that PC and its components demonstrated strong potential for inhibiting BChE, in comparison to AChE inhibition. Furthermore, PC exhibited potent and highly selective inhibition of BChE activity, with a selectivity index of BChE > 20, likely attributed to the anti-BChE activity of ZO, as shown in the equivalence assay.

While many current AD treatments focus on AChE inhibition, the high BChE selectivity of PC presents a notable advantage. Conventional AChE inhibitors, such as donepezil, galantamine, and rivastigmine, generally exhibit moderate SI values for BChE, typically less than 10 [35]. In the realm of natural products, BChE-selective inhibition remains relatively rare. A comprehensive review of plant-based BChE inhibitors reveals that most natural extracts or isolated compounds typically exhibit SI values under 10 [36]. For example, β-carboline and quinoline alkaloids derivatives from the plants of genus *Peganum* showed BChE SI values between 0.19 and 10.82 [37]. In this context, the observed BChE SI of greater than 20 for PC highlights a remarkably high degree of selectivity, positioning it as a potentially promising natural agent for targeting BChE. Nonetheless, it is important to note that in vitro SI values may not fully predict in vivo efficacy due to complex pharmacokinetic and metabolic factors. Therefore, further in vivo behavioral models and pharmacodynamic profiling are essential to conclusively confirm the therapeutic relevance of this observed selectivity.

Furthermore, the limitation of AChE inhibitors is their long-term use in clinical trials, in which unwanted side effects such as nausea and vomiting can develop through the inhibition of peripheral cholinesterase [35]. In contrast, inhibiting BChE does not lead to these peripheral adverse effects [38]. Moreover, the effectiveness of AChE inhibitors tends to diminish over time due to the progressive reduction of acetylcholine in the advanced stages of AD, while levels of BChE increase [39]. Thus, the increasing levels of BChE in advanced AD raise concerns about the efficacy of AChE-selective inhibitors [40,41,42], as BChE acts as a compensatory enzyme when AChE activity declines during disease progression. Consequently, selective BChE inhibitors may represent a significant advancement in AD treatment, as they can increase acetylcholine levels in the brain while reducing peripheral side effects [43]. This positions the high BChE selectivity of PC as a promising therapeutic strategy.

Meanwhile, the other AD pathology is Aβ aggregation, a key event in the AD progression. Therefore, Aβ aggregates are also considered a promising target for AD treatment. PC demonstrated the ability to inhibit Aβ aggregation, with OI showing the greatest potential in contributing to this activity within the PC formula. As mentioned earlier, OI served as the primary herbal plant responsible for the multitarget actions of PC, due to its higher concentration compared to the other components.

The moderate concentration of H_2_O_2_ can induce DNA fragmentation and morphological alterations, leading to apoptosis [44]. Our findings demonstrated that PC at a concentration of 100 µM effectively mitigated oxidative damage from H_2_O_2_. This result suggested that OI is the most potent component of PC, contributing to all the activities observed in the formula. OI exhibited a neuroprotective effect at the same 100 µM concentration as PC, while the other components began to protect cells at lower concentrations. However, since OI was present in higher concentrations in the formula, it was likely the key factor responsible for the neuroprotective effect of PC at the 100 µM concentration.

Furthermore, network pharmacology analysis was used to explore the other key targets and pathways associated with the PC formula in AD. The analysis of the PPI network provided valuable insights into the therapeutic potential of the major active components of the PC formula and identified several core targets that are significantly involved in AD, including APP, GAPDH, CASP3, PTGS2, GSK3B, and PPARG. These genes are associated with oxidative stress, inflammation, Aβ and tau pathology, all of which play a crucial role in AD progression.

The key pathological features of AD include the accumulation of extracellular Aβ plaques and intracellular neurofibrillary tangles composed of hyperphosphorylated tau protein. These abnormalities contribute to neuronal death, leading to progressive memory impairment and cognitive decline [45,46]. Amyloid precursor protein or APP plays a key role in the formation of Aβ plaques, a hallmark of AD pathology. APP mutations caused the excessive APP cleavage, leading to increase Aβ production and promote its aggregation [47]. Therefore, APP dysregulation contributes to neurodegeneration. Glycogen synthase kinase 3 beta (GSK3B) is the predominant isoform of GSK in the central nervous system. Studies have shown that GSK3B is hyperactive in the AD brain, playing a crucial role in tau phosphorylation, which leads to the formation of neurofibrillary tangles [48,49].

Extensive research has identified oxidative stress as one of the key hallmarks of AD pathology. Oxidative modifications contribute to the loss of neurons and synapses, ultimately impairing normal brain function. Also, the neuronal death in AD can be primarily associated with apoptosis, which involves caspase signaling and DNA fragmentation. Caspases are cysteine-dependent, aspartate-specific proteases that recognize and cleave substrates at DXXD motifs. Upstream caspases, such as caspase-8 or -9, activate downstream effectors like caspase-3 and -7, leading to the degradation of cellular proteins. Glyceraldehyde-3-phosphate dehydrogenase (GAPDH) enzymes are a widely expressed group of oxidoreductases primarily involved in glucose metabolism. GAPDH has served as a housekeeping protein or gene in studies, which has been commonly used as a loading control in Western blot analyses due to its highly conserved gene and protein sequences across different species [50]. The neuronal apoptosis in AD alters GAPDH oxidation, leading to oxidative dysfunction of GAPDH. This impairment may play a significant role in the loss of neuronal function and contribute to neurodegeneration in the AD brain [50,51,52,53]. Therefore, GAPDH and caspase-3 are involved in neuronal apoptosis and oxidative stress, processes central to the progression of AD.

Chronic neuroinflammation is another prominent hallmark of the AD brain. Amyloid plaques are surrounded by activated microglia and astrocytes, which play a crucial role in the inflammatory response [54]. Upon activation, microglia release inflammatory cytokines and chemokines such as interleukin (IL)-1β, IL-6, monocyte chemotactic protein-1 (MCP-1), and tumor necrosis factor (TNF)-α. This prolonged activation and production of inflammatory cytokines are believed to exacerbate disease progression and directly contribute to neuronal loss [54,55]. The PTGS2 gene, also known as the cyclooxygenase-2 (COX-2) gene, is widely expressed in inflammatory cells within the central nervous system [56]. Cyclooxygenases are key enzymes that convert arachidonic acid into eicosanoids, classical mediators of inflammation that contribute to neurodegeneration [57]. Elevated PTGS2 levels are observed in hippocampal pyramidal neurons of AD patients [58,59]. Moreover, polymorphisms in the PTGS2 gene could increase susceptibility to AD, linking inflammation and arachidonic acid metabolism to AD pathology [56]. Lastly, PPARG or peroxisome proliferator-activated receptor gamma regulates the critical metabolism of lipid and carbohydrate. The activation of PPARG is associated with the regulation of endocrine factors, which has led to the development of specific PPARG agonists for the treatment of type II diabetes [60]. Moreover, PPARG has the ability to suppress the expression of proinflammatory genes in myeloid lineage cells, such as microglia and macrophages. This action may prove beneficial in the treatment of chronic neuroinflammatory diseases [60,61,62]. Thus, PTGS2 and PPARG are key players in the inflammatory response, which linked to the neuroinflammation observed in AD.

These genes are integral to the molecular mechanisms of AD, and their modulation by the PC formula highlights its potential as a therapeutic strategy. It is crucial to emphasize that network pharmacology in this context serves as a powerful tool for hypothesis generation, providing insights into potential mechanisms and clinical applications of the PC formula in AD. However, these findings are based solely on expression data from public databases. Therefore, further experimental confirmation of these potential targets, for instance, through techniques like qPCR or Western blot, is essential to validate these computationally derived hypotheses.

Collectively, the growing recognition of AD as a multifactorial disorder has led to increased interest in multitarget therapeutic strategies [63,64,65]. Our study provides evidence that combining these specific Thai herbs in the PC formula can produce synergistic effects, thereby enhancing overall efficacy against multiple AD-related pathological processes. However, further research is essential to comprehensively assess the safety and toxicity profile of this polyherbal formulation and to definitively confirm its precise mechanisms of action in vivo and in clinical settings.

Importantly, these findings reflect a broader trend in neurodegenerative research: the shift from single-target agents to multitarget therapeutics capable of modulating complex pathophysiological networks. As AD is increasingly recognized as a systems-level disorder, polyherbal formulations like the PC formula—derived from traditional medical knowledge and validated through modern techniques—represent a promising frontier in integrative drug development.

## 4. Materials and Methods

### 4.1. In Vitro Experimental Validation

#### 4.1.1. Formula Preparation

The PC formula consists of three components: (i) the fruit of *Oroxylum indicum* (OI), (ii) the rhizome of *Zingiber officinale* (ZO), and (iii) the rhizome of *Boesenbergia rotunda* (BR). Both the pre-mixed PC formula powder and individual plant component powders were sourced from Chao Phya Abhaibhubejhr Hospital Foundation in Prachinburi Province, Thailand. Ms. Benjawan Leenin, Chief of the Traditional Knowledge Center at Chao Phya Abhaibhubejhr Hospital Foundation, performed the botanical identification. Voucher specimens for OI, ZO, and BR are deposited at the Chao Phya Abhaibhubejhr Hospital museum under accession numbers ABH37, ABH39, and ABH38, respectively. Crude extracts of PC, OI, ZO, and BR were prepared by macerating the powders with 95% ethanol at a 1:5 (*w*/*v*) ratio for 7 d. The ethanolic extract was filtered and concentrated using a rotary evaporator at 55 °C, and then freeze-dried. The dried extract was then kept in airtight containers at 4 °C.

#### 4.1.2. Identification of PC Formula Components by HPLC

The components of the PC formula were identified using High-Performance Liquid Chromatography (HPLC) on an Agilent 1260 Infinity II system equipped with a 1260 vial sampler. Separation of seven active compounds was achieved using a HiQsil C18 column (250 mm × 4.6 mm, 5 µm) with gradient elution. The mobile phase consisted of 1% formic acid (A) and 10% methanol in acetonitrile (B), delivered at a constant flow rate of 0.5 mL/min. The gradient program ran from 70% A to 5% A over 30 min. The column temperature was maintained at 40 °C, and the injection volume was 10 µL. UV detection was performed at 285 nm, following a method adapted from Rojsanga et al. [31].

#### 4.1.3. Investigation of Total Phenolic Content (TPC) and Total Flavonoid Contents (TFC)

The Folin–Ciocalteu (FC) method was used to assess TPC. Briefly, 7.5% (*w*/*v*) Na_2_CO_3_ (75 µL) (Unilab Pharmaceuticals, Bangkok, Thailand) was added after PC formula or each plant (10 µL) was mixed with FC reagent (75 µL) (Sigma-Aldrich, SM Chemical supplies Co., Ltd., Bangkok, Thailand) and left in dark for 5 min. The absorbance was measured at 700 nm after 2 h. Gallic acid (Sigma-Aldrich, SM Chemical supplies Co., Ltd., Bangkok, Thailand) was used as the standard to generate a calibration curve. The TPC of each extract is expressed in milligrams of gallic equivalents per gram of crude extract (mg GAE/g crude extract). AlCl_3_ colorimetric method was used to determine TFC [8]. Briefly, PC formula or each plant (20 µL) was mixed with 2.5% (*w*/*v*) AlCl_3_ (15 µL) (Sigma-Aldrich, SM Chemical supplies Co., Ltd., Bangkok, Thailand), CH_3_COONa (100 g/L, 20 µL) (Sigma-Aldrich, SM Chemical supplies Co., Ltd., Bangkok, Thailand), and distilled water (145 µL). After 15 min, the absorbance was measured at 450 nm. Quercetin (Sigma-Aldrich, SM Chemical supplies Co., Ltd., Bangkok, Thailand) was used as a reference standard for the calibration curve. The TFC of each extract is expressed as milligrams of quercetin equivalents per gram of crude extract (mg QE/g crude extract).

#### 4.1.4. Investigation of Antioxidant Activities Using Diphenyl-Picrylhydrazyl (DPPH), and 2, 2’-Azinobis-(3-Ethylbenzothiazoline-6-Sulfonic Acid) (ABTS) Assay

For the DPPH assay, PC formula or each plant (50 µL) was incubated with 0.2 mM DPPH (50 µL) (Sigma-Aldrich, SM Chemical supplies Co., Ltd., Bangkok, Thailand) for 30 min, and the absorbance was measured at 550 nm following incubation. For ABTS assay, the stock ABTS solution was prepared by incubating 7 mM ABTS (Sigma-Aldrich, SM Chemical supplies Co., Ltd., Bangkok, Thailand) with 2.45 mM K_2_S_2_O_8_ for 12 h in the dark at room temperature. The solution was then diluted by ethanol to obtain an absorbance of 0.70 ± 0.02 at 700 nm for the working ABTS solution. PC formula or each plant (50 µL) was mixed with ABTS working solution (100 µL), and the absorbance was measured at 700 nm after 15 min [10].

#### 4.1.5. Investigation of Cholinesterase Inhibitory Activity

Acetylcholinesterase (AChE) and butyrylcholinesterase (BChE) activities were determined using a slightly modified method [66]. Briefly, PC formula or each plant (25 µL) was incubated with 1 mM ATCI or BTCI (25 µL), 0.1 M PBS (50 µL), 1 mM DTNB (125 µL), and AChE from electric eel type VI-S (Sigma-Aldrich, SM Chemical supplies Co., Ltd., Bangkok, Thailand) or BChE from equine serum (50 µL) (Sigma-Aldrich, SM Chemical supplies Co., Ltd., Bangkok, Thailand). The reaction was monitored every 30 s for 5 min at 405 nm.

#### 4.1.6. Investigation of Amyloid Beta (Aβ) Aggregation Inhibition

Thioflavin T (ThT) assay was used to detect Aβ aggregation [7]. Briefly, PC formula and each plant (5 µL) was mixed with 10 µM of Aβ_1–42_ (20 µL) for 48 h in dark. After that, 5 µM ThT (175 µL) in glycine/NaOH buffer pH 8.5 was added and measured the fluorescence intensity at 446 nm for excitation and 490 nm for emission.

#### 4.1.7. Cytotoxicity and Neuroprotective Effect on Hydrogen Peroxide (H_2_O_2_)-Induced Cell Damage

The SH-SY5Y cells were cultured in DMEM/Ham’s F-12 (Sigma-Aldrich, SM Chemical supplies Co., Ltd., Bangkok, Thailand) and completed with 10% FBS, and penicillin–streptomycin. The cells with a density of 5 × 10^5^ cells/mL were seeded into 96-well plate prior to treatment. For the cytotoxicity test, PC formula or each plant at various concentration was incubated with the cells for 2 h and then the cell viability was determined. Next, to evaluate the neuroprotective effect, cells were treated with PC formula or each plant at various concentrations for 2 h. After removing the culture media, H_2_O_2_ at the concentration of 400 μM was added to induce oxidative damage, and the cells were incubated for an additional 2 h before assessing cell viability [44]. Cell viability was determined using MTT (3-(4, 5-dimethyl-2-thiazolyl)-2, 5-diphenyl-2H-tetrazolium bromide) assay, with absorbance measured at 550 nm [6].

### 4.2. Pharmacology Network Analysis

#### 4.2.1. Collection of PC Formula Targets and the Identification of AD Potential Targets

The SwissTargetPrediction database (http://www.swisstargetprediction.ch/) [67], accessed on 2 March 2025, was employed as a web server for identifying potential targets of the main phytochemicals in the PC formula. The canonical SMILES of these compounds were input into the SwissTargetPrediction, with the target species specified as *Homo sapiens*. To identify potential AD targets, GeneCards (https://www.genecards.org), accessed on 15 March 2025, with a relevance score greater than 3 and DisGeNET (https://www.disgenet.org/), accessed on 15 March 2025, were used, searching with the keyword “Alzheimer’s disease.”

#### 4.2.2. Protein–Protein Interaction Network Construction and Screening of Core Targets

A Protein–Protein Interaction (PPI) network of the target proteins was constructed using the STRING database (version 12.0, https://string-db.org/) [68], accessed on 28 March 2025, and visualized with Cytoscape software (version 3.10.1) [69]. PPIs with an interaction score above the threshold of 0.4 (medium confidence) were considered. In the PPI network, the degree represents the number of directly connected nodes to a given node. Core targets are defined by their degree values, calculated through Network Analysis, a Cytoscape plugin. The darker the red color of a node, the more significant its role in the PPI network. Additionally, three methods from the cytoHubba plugin including degree, betweenness, and closeness centrality, were applied to identify the top ten hub proteins [70]. Hub genes were ranked based on their proximity and their overall interaction with the entire network. Finally, common hub proteins were determined by intersecting the results from the cytoHubba methods.

#### 4.2.3. Enrichment Analysis of Gene Ontology, and the Kyoto Encyclopedia of Genes

Gene ontology (GO) terms are classified into three categories: biological process (BP), cellular component (CC), and molecular function (MF). GO and KEGG pathway enrichment analyses were conducted using DAVID (https://davidbioinformatics.nih.gov/), accessed on 28 March 2025, with “*Homo sapiens*” as the species [71,72]. Statistical significance was considered when the *p*-value was less than 0.05. The top 5 GO terms and KEGG pathways, sorted by *p*-value, were visualized using an online tool (http://www.bioinformatics.com.cn/), accessed on 28 March 2025.

#### 4.2.4. Validation Analysis of Target Genes Correlated with AD

The AlzData database (http://www.alzdata.org/), accessed on 30 March 2025, a widely utilized Alzheimer’s disease (AD) database that compiles current high-throughput omics data [73], was employed to analyze the connection between the PC formula targets and AD. The normalized expression levels of the PC formula targets associated with AD in both the control and AD groups from the Gene Expression Omnibus (GEO) data set were examined using the “Differential expression” module of AlzData to evaluate any changes in targets within key signaling pathways.

### 4.3. Statistical Analysis

All data are presented as mean ± SD. For the cell culture experiments, statistical analysis was performed using one-way ANOVA, followed by Tukey’s post hoc test. For other experiments, the Kruskal–Wallis test followed by pairwise Mann–Whitney U tests was used to assess group differences. A *p*-value < 0.05 was considered statistically significant.

## 5. Conclusions

This study provides preliminary scientific support for the Pheka capsule (PC) formula, a traditional Thai polyherbal medicine, as a promising multi-target candidate for further research into Alzheimer’s disease (AD). The demonstrated in vitro activities, encompassing antioxidant, selective BChE inhibitory, anti-Aβ aggregation, and neuroprotective effects, align with the complex pathophysiology of AD. Our network pharmacology analysis provides a valuable framework for hypothesis generation by identifying potential mechanisms of action and highlighting key AD-related targets like APP, GAPDH, CASP3, PTGS2, GSK3B, and PPARG, along with relevant modulated pathways. This analysis suggests how the bioactive compounds in the PC formula might exert their effects. The synergistic potential of combining *Oroxylum indicum* (OI), *Zingiber officinale* (ZO), and *Boesenbergia rotunda* (BR) highlights the value of exploring traditional polyherbal formulations for complex diseases. While these computational findings are insightful, it is important to note that they are based solely on expression data from public databases. Therefore, the identified targets and pathways require further experimental validation. Future research should prioritize comprehensive in vivo investigations, including safety and toxicity assessments and, ultimately, clinical trials to validate its efficacy in treating Alzheimer’s disease. This work contributes to the growing interest in multitarget interventions for neurodegenerative disorders and underscores the importance of integrating traditional knowledge with modern scientific approaches in drug discovery.

## Figures and Tables

**Table 1:** TPC and TFC of the PC formula and its components.

Extracts	Total Phenolic Content (mg Gallic Acid Equivalent/g Extract)	Total Flavonoid Content (mg Quercetin Equivalent/g Extract)
PC formula	73.65 ± 1.39 ^b^	31.86 ± 1.13 ^b^
OI	62.58 ± 1.22 ^a^	14.83 ± 0.65 ^a^
ZO	205.46 ± 7.65 ^c^	110.48 ± 4.06 ^c^
BR	83.29 ± 5.96 ^b^	131.81 ± 3.22 ^d^

^a,b,c,d^ Means in a row with different superscript letters are significantly different based on the Kruskal–Wallis test followed by pairwise Mann–Whitney U tests (*p* < 0.05).

**Table 2:** IC_50_ values (µg/mL) from DPPH and ABTS assays for PC formula and its plant constituents.

Samples	DPPH (IC_50_, µg/mL)	ABTS (IC_50_, µg/mL)
PC formula	54.71 ± 4.15 ^d^	86.67 ± 2.66 ^d^
OI	66.96 ± 3.35 ^c^	95.14 ± 1.32 ^e^
ZO	27.99 ± 2.79 ^b^	24.01 ± 1.05 ^a^
BR	180.43 ± 4.16 ^e^	58.17 ± 0.90 ^c^
Trolox (µM)	19.87 ± 1.53 ^a^	40.18 ± 1.36 ^b^

^a,b,c,d,e^ Means in a row with different superscript letters are significantly different based on the Kruskal–Wallis test followed by pairwise Mann–Whitney U tests (*p* < 0.05).

**Table 3:** The inhibition of AChE and BChE (IC_50_; µg/mL) of PC formula and its plant constituents.

Samples	IC_50_ (µg/mL) of ChE		Selectivity	
	AChE	BChE	AChE	BChE
PC formula	2596.94 ± 0.50 ^d^	105.18 ± 5.43 ^c^	0.04	24.69
OI	2122.70 ± 0.19 ^d^	239.49 ± 3.13 ^e^	0.11	8.86
ZO	1026.59 ± 0.08 ^b^	59.52 ± 1.13 ^b^	0.06	17.42
BR	1787.51 ± 0.01 ^c^	178.47 ± 3.38 ^d^	0.10	10.02
Tacrine (µM)	0.25 ± 0.02 ^a^	0.0051 ± 0.0002 ^a^	0.02	48.76

^a,b,c,d,e^ Means in a row with different superscript letters are significantly different based on the Kruskal–Wallis test followed by pairwise Mann–Whitney U tests (*p* < 0.05).

**Table 4:** Inhibition of Aβ aggregation of PC formula and each individual plant (IC_50_; µg/mL).

Extracts and Standard	Beta Amyloid Aggregation (IC_50_, µg/mL)
PC formula	20.60 ± 2.14 ^b^
OI	47.73 ± 1.06 ^c^
ZO	20.71 ± 1.60 ^b^
BR	>2000 ^d^
Curcumin (µM)	14.56 ± 0.50 ^a^

^a,b,c,d^ Means in a row with different superscript letters are significantly different based on the Kruskal–Wallis test followed by pairwise Mann–Whitney U tests (*p* < 0.05).

## Data Availability

The data generated in the present study may be requested from the corresponding author.

## Supplementary Materials

The following supporting information can be downloaded at: https://www.mdpi.com/article/10.3390/ijms26136287/s1.

## References

[1] (2024). Alzheimer’s Association Report. Alzheimer’s disease facts and figures. Alzheimer’s Dement. J. Alzheimer’s Assoc..

[2] Wong W. (2020). Economic burden of Alzheimer disease and managed care considerations. Am. J. Manag. Care.

[3] Monteiro A.R., Barbosa D.J., Remião F., Silva R. (2023). Alzheimer’s disease: Insights and new prospects in disease pathophysiology, biomarkers and disease-modifying drugs. Biochem. Pharmacol..

[4] Peng Y., Jin H., Xue Y., Chen Q., Yao S., Du M., Liu S. (2023). Current and future therapeutic strategies for Alzheimer’s disease: An overview of drug development bottlenecks. Front. Aging Neurosci..

[5] Breijyeh Z., Karaman R. (2020). Comprehensive Review on Alzheimer’s Disease: Causes and Treatment. Molecules.

[6] Chheng C., Waiwut P., Plekratoke K., Chulikhit Y., Daodee S., Monthakantirat O., Pitiporn S., Musigavong N., Kwankhao P., Boonyarat C. (2020). Multitarget Activities of Kleeb Bua Daeng, a Thai Traditional Herbal Formula, Against Alzheimers Disease. Pharmaceuticals.

[7] Boonyarat C., Yenjai C., Monthakantirat O., Kaewamatawong R., Poonsawas P., Wangboonskul J., Chaiwiwatrakul S., Waiwut P. (2022). Multifunctionality of Clausena harmandiana Extract and Its Active Constituents against Alzheimer’s Disease. Curr. Issues Mol. Biolology.

[8] Summat R., Waiwut P., Daodee S., Nualkaew N., Phemphunananchai K., Arsito P.N., Chulikhit Y., Montakantirat O., Khamphukdee C., Boonyarat C. (2025). Phytomedicine Potential of *Oroxylum indicum* Root and Its Constituents: Targeting Alzheimer’s Disease. Plants.

[9] Arsito N.V., Waiwut P., Yenjai C., Arthan S., Monthakantirat O., Nualkaew N., Takomthong P., Boonyarat C. (2023). Multifunctional effect of flavonoids from Millettia brandisiana against Alzheimer’s disease pathogenesis. Heliyon.

[10] Takomthong P., Waiwut P., Yenjai C., Wangboonskul J., Plekratoke K., Arsito N.V., Ballatore C., Boonyarat C. (2025). Kaempferia parviflora extract and its methoxyflavones as potential anti-Alzheimer assessing In Vitro, Integrated Computational Approach, and In Vivo Impact on behaviour in Scopolamine-Induced Amnesic Mice. PLoS ONE.

[11] Yoo K., Park S. (2012). Terpenoids as Potential Anti-Alzheimer’s Disease Therapeutics. Molecules.

[12] He K. (2015). Traditional Chinese and Thai medicine in a comparative perspective. Complement. Ther. Med..

[13] Pimpa R. (2022). Health Promotion in Thai Traditional Medicine Practice: Related Theories and Strategies. J. Thai Tradit. Altern. Med..

[14] Vadhnapijyakul A., Suttipanta N. (2014). The Promotion of Thai Traditional Medicine Policy in Government Hospitals: Myth or Reality. Isan J. Pharm. Sci. IJPS.

[15] Che C.-T., Wang Z.J., Chow M.S.S., Lam C.W.K. (2013). Herb-Herb Combination for Therapeutic Enhancement and Advancement: Theory, Practice and Future Perspectives. Molecules.

[16] Aryal B., Raut B.K., Bhattarai S., Bhandari S., Tandan P., Gyawali K., Sharma K., Ranabhat D., Thapa R., Aryal D. (2022). Potential Therapeutic Applications of Plant-Derived Alkaloids against Inflammatory and Neurodegenerative Diseases. Evid. -Based Complement. Altern. Med..

[17] Rizvi S.A.A., Einstein G.P., Tulp O.L., Sainvil F., Branly R. (2022). Introduction to Traditional Medicine and Their Role in Prevention and Treatment of Emerging and Re-Emerging Diseases. Biomolecules.

[18] Pitiporn S., Tengtermwong N. (2016). List of Herbal Medicine Products, Chao Phya Abhaibhubejhr Hospital.

[19] Sinphurmsukskul S., Sangwatanaroj S. (2019). The effect of Paeka capsules on LDL cholesterol levels in patients with hypercholesterolemia. Chula J. Intern. Med..

[20] Mairuae N., Connor J.R., Buranrat B., Lee S.Y. (2019). *Oroxylum indicum* (L.) extract protects human neuroblastoma SH-SY5Y cells against β-amyloid-induced cell injury. Mol. Med. Rep..

[21] Mairuae N., Cheepsunthorn P., Buranrat B. (2022). Antioxidant and anti-inflammatory activities of *Oroxylum indicum* Kurz (L.) fruit extract in lipopolysaccharide-stimulated BV2 microglial cells. Trop. J. Pharm. Res..

[22] Li Y., Hong Y., Han Y., Wang Y., Xia L. (2016). Chemical characterization and antioxidant activities comparison in fresh, dried, stir-frying and carbonized ginger. J. Chromatogr. B.

[23] Talebi M., İlgün S., Ebrahimi V., Talebi M., Farkhondeh T., Ebrahimi H., Samarghandian S. (2021). *Zingiber officinale* ameliorates Alzheimer’s disease and Cognitive Impairments: Lessons from preclinical studies. Biomed. Pharmacother..

[24] Tung B.T., Thu D.K., Thu N.T.K., Hai N.T. (2017). Antioxidant and acetylcholinesterase inhibitory activities of ginger root (*Zingiber officinale* Roscoe) extract. J. Complement. Integr. Med..

[25] Zhang S., Mak K.-K., Marappan P., Balijepalli M.K., Chong G.-H., Rao Pichika M. (2022). Simple HPLC Method for the Quantification of Gingerols (4-, 6-, 8-, and 10-) and Shogaols (6-, 8-, AND 10-) in *Zingiber officinale* var. Rubrum Supercritical Carbon Dioxide (SC-CO2) Extract. Rasayan J. Chem..

[26] Ho S.-C., Chang K.-S., Lin C.-C. (2013). Anti-neuroinflammatory capacity of fresh ginger is attributed mainly to 10-gingerol. Food Chem..

[27] Isa N.M., Abdelwahab S.I., Mohan S., Abdul A.B., Sukari M.A., Taha M.M.E., Syam S., Narrima P., Cheah S.C., Ahmad S. (2012). In vitro anti-inflammatory, cytotoxic and antioxidant activities of boesenbergin A, a chalcone isolated from *Boesenbergia rotunda* (L.) (fingerroot). Braz. J. Med. Biol. Res..

[28] Zhang Y., Chen H., Li R., Sterling K., Song W. (2023). Amyloid β-based therapy for Alzheimer’s disease: Challenges, successes and future. Signal Transduct. Target. Ther..

[29] Panomai P., Thapphasaraphong S., Nualkaew N. (2024). A Comparative Study of Two *Oroxylum indicum* (L.) Kurz. Phenotypes Based on Phytochemicals and Antioxidant Effects, and the Anti-Inflammatory Activity of Leaf and Pod Extracts. Plants.

[30] Pattamadilok D., Sakpetch A. (2021). Isolation of Pinostrobin, a Chemical Marker from Fingerroots for Quality Control Purposes. J. Thai Tradit. Altern. Med..

[31] Rojsanga P., Schwaiger S., Stuppner H., Sithisarn P. (2023). Determination of Phytochemical Contents in Extracts from Different Growth Stages of *Oroxylum indicum* Fruits Using HPLC-DAD and QAMS Methods. Molecules.

[32] Tabboon P., Tantiyasawasdikul S., Sripanidkulchai B. (2021). Development of simultaneous determination of five active compounds of ginger by high performance liquid chromatographic method. Isan J. Pharm. Sci..

[33] Tan B.C., Tan S.K., Wong S.M., Ata N., Rahman N.A., Khalid N. (2015). Distribution of Flavonoids and Cyclohexenyl Chalcone Derivatives in Conventional Propagated and In Vitro-Derived Field-Grown *Boesenbergia rotunda* (L.) *Mansf*.. Evid.-Based Complement. Altern. Med. Ecam.

[34] Tohma H., Gülçin İ., Bursal E., Gören A.C., Alwasel S.H., Köksal E. (2017). Antioxidant activity and phenolic compounds of ginger (*Zingiber officinale* Rosc.) determined by HPLC-MS/MS. J. Food Meas. Charact..

[35] Marucci G., Buccioni M., Ben D.D., Lambertucci C., Volpini R., Amenta F. (2021). Efficacy of acetylcholinesterase inhibitors in Alzheimer’s disease. Neuropharmacology.

[36] Orhan I., Şener B., Choudhary M.I., Khalid A. (2004). Acetylcholinesterase and butyrylcholinesterase inhibitory activity of some Turkish medicinal plants. J. Ethnopharmacol..

[37] Zhao T., Ding K., Zhang L., Cheng X., Wang C., Wang Z. (2013). Acetylcholinesterase and butyrylcholinesterase inhibitory activities of β-carboline and quinoline alkaloids derivatives from the plants of genus Peganum. J. Chem..

[38] Žužek M.C. (2024). Advances in cholinesterase inhibitor research—An overview of preclinical studies of selected organoruthenium(II) complexes. Int. J. Mol. Sci..

[39] Chen Z., Huang J., Yang S., Hong F. (2022). Role of cholinergic signaling in Alzheimer’s disease. Molecules.

[40] Atatreh N., Al Rawashdah S., Al Neyadi S.S., Abuhamdah S.M., Ghattas M.A. (2019). Discovery of new butyrylcholinesterase inhibitors via structure-based virtual screening. J. Enzym. Inhib. Med. Chem..

[41] Lane R.M., Potkin S.G., Enz A. (2006). Targeting acetylcholinesterase and butyrylcholinesterase in dementia. Int. J. Neuropsychopharmacol..

[42] Mushtaq G., Greig N.H., Khan J.A., Kamal M.A. (2014). Status of Acetylcholinesterase and Butyrylcholinesterase in Alzheimer’s Disease and Type 2 Diabetes Mellitus. CNS Neurol. Disord. Drug Targets.

[43] Košak U., Brus B., Knez D., Šink R., Žakelj S., Trontelj J., Pišlar A., Šlenc J., Gobec M., Živin M. (2016). Development of an in-vivo active reversible butyrylcholinesterase inhibitor. Sci. Rep..

[44] Moon H., Yun J. (2023). Neuroprotective effects of hesperetin on H_2_O_2_-induced damage in neuroblastoma SH-SY5Y cells. Nutr. Res. Pract..

[45] DeTure M.A., Dickson D.W. (2019). The neuropathological diagnosis of Alzheimer’s disease. Mol. Neurodegener..

[46] Kent S.A., Spires-Jones T.L., Durrant C.S. (2020). The physiological roles of tau and Aβ: Implications for Alzheimer’s disease pathology and therapeutics. Acta Neuropathol..

[47] Zhang Y., Thompson R., Zhang H., Xu H. (2011). APP processing in Alzheimer’s disease. Mol. Brain.

[48] Simone A.D., Tumiatti V., Andrisano V., Milelli A. (2021). Glycogen Synthase Kinase 3β: A new gold rush in anti-Alzheimer’s disease multitarget drug discovery?. J. Med. Chem..

[49] Lauretti E., Dincer O., Praticò D. (2020). Glycogen synthase kinase-3 signaling in Alzheimer’s disease. Biochim. Biophys. Acta Mol. Cell Res..

[50] Butterfield D.A., Hardas S.S., Bader Lange M.L. (2010). Oxidatively modified glyceraldehyde-3-phosphate dehydrogenase (GAPDH) and Alzheimer disease: Many pathways to neurodegeneration. J. Alzheimer’s Dis.: JAD.

[51] Itakura M., Nakajima H., Kubo T., Semi Y., Kume S., Higashida S., Kaneshige A., Kuwamura M., Harada N., Kita A. (2015). Glyceraldehyde-3-phosphate Dehydrogenase Aggregates Accelerate Amyloid-β Amyloidogenesis in Alzheimer Disease. J. Biol. Chem..

[52] Cornett K., Puderbaugh A., Back O., Craven R. (2022). GAPDH in neuroblastoma: Functions in metabolism and survival. Front. Oncol..

[53] Tsai C.W., Tsai C.F., Lin K.H., Chen W.J., Lin M.S., Hsieh C.C., Lin C.C. (2020). An investigation of the correlation between the S-glutathionylated GAPDH levels in blood and Alzheimer’s disease progression. PLoS ONE.

[54] Sobue A., Komine O., Yamanaka K. (2023). Neuroinflammation in Alzheimer’s disease: Microglial signature and their relevance to disease. Inflamm. Regen..

[55] Twarowski B., Herbet M. (2023). Inflammatory processes in Alzheimer’s disease—Pathomechanism, diagnosis and treatment: A review. Int. J. Mol. Sci..

[56] Chen Q., Liang B., Wang Z., Cheng X., Huang Y., Liu Y., Huang Z. (2016). Influence of four polymorphisms in ABCA1 and PTGS2 genes on risk of Alzheimer’s disease: A meta-analysis. Neurol. Sci..

[57] Moussa N., Dayoub N. (2023). Exploring the role of COX-2 in Alzheimer’s disease: Potential therapeutic implications of COX-2 inhibitors. Saudi Pharm. J. SPJ.

[58] Guan P., Wang P. (2019). Integrated communications between cyclooxygenase-2 and Alzheimer’s disease. FASEB J..

[59] Michele S., Salluzzo M.G., Calogero A.E., Raffaele F., Bosco P. (2014). Association study of COX-2 (PTGS2) –765 G/C promoter polymorphism by pyrosequencing in Sicilian patients with Alzheimer’s disease. Arch. Med. Sci..

[60] Govindarajulu M., Pinky P.D., Bloemer J., Ghanei N., Suppiramaniam V., Amin R. (2018). Signaling mechanisms of selective PPARγ modulators in Alzheimer’s disease. PPAR Res..

[61] Heneka M.T., Reyes-Irisarri E., Hüll M., Kummer M.P. (2011). Impact and Therapeutic Potential of PPARs in Alzheimer’s Disease. Curr. Neuropharmacol..

[62] Strosznajder A.K., Wójtowicz S., Jeżyna M.J., Sun G.Y., Strosznajder J.B. (2021). Recent insights on the role of PPAR-β/δ in neuroinflammation and neurodegeneration, and its potential target for therapy. Neuromolecular Med..

[63] Turgutalp B., Kizil C. (2024). Multi-target drugs for Alzheimer’s disease. Trends Pharmacol. Sci..

[64] Xiao D., Zhang C. (2024). Current therapeutics for Alzheimer’s disease and clinical trials. Explor. Neurosci..

[65] Zhang L., Jiang Q., Wang X., Jaisi A., Olatunji O.J. (2023). *Boesenbergia rotunda* displayed anti-inflammatory, antioxidant and anti-apoptotic efficacy in doxorubicin-induced cardiotoxicity in rats. Sci. Rep..

[66] Ellman G.L., Courtney K.D., Andres V.J., Feather-stone R.M. (1961). A new and rapid colorimetric determination of acetylcholinesterase activity. Biochem Pharmacol..

[67] Daina A., Michielin O., Zoete V. (2019). SwissTargetPrediction: Updated data and new features for efficient prediction of protein targets of small molecules. Nucleic Acids Res..

[68] Szklarczyk D., Kirsch R., Koutrouli M., Nastou K., Mehryary F., Hachilif R., Gable A.L., Fang T., Doncheva N.T., Pyysalo S. (2023). The STRING database in 2023: Protein-protein association networks and functional enrichment analyses for any sequenced genome of interest. Nucleic Acids Res..

[69] Shannon P., Markiel A., Ozier O., Baliga N.S., Wang J.T., Ramage D., Amin N., Schwikowski B., Ideker T. (2003). Cytoscape: A software environment for integrated models of biomolecular interaction networks. Genome Res..

[70] Chin C.-H., Chen S.-H., Wu H.-H., Ho C.-W., Ko M.-T., Lin C.-Y. (2014). cytoHubba: Identifying hub objects and sub-networks from complex interactome. BMC Syst. Biol..

[71] Huang D.W., Sherman B.T., Lempicki R.A. (2009). Systematic and integrative analysis of large gene lists using DAVID bioinformatics resources. Nat. Protoc..

[72] Sherman B.T., Hao M., Qiu J., Jiao X., Baseler M.W., Lane H.C., Imamichi T., Chang W. (2022). DAVID: A web server for functional enrichment analysis and functional annotation of gene lists (2021 update). Nucleic Acids Res..

[73] Xu M., Zhang D., Luo R., Wu Y., Zhou H., Kong L., Bi R., Yao Y. (2018). A systematic integrated analysis of brain expression profiles reveals YAP1 and other prioritized hub genes as important upstream regulators in Alzheimer’s disease. Alzheimer’s Dement..

