# Supplementary figures and images for: Pharmacological Evaluation of a Traditional Thai Polyherbal Formula for Alzheimer’s Disease: Evidence from In Vitro and In Silico Studies
Source: Int J Mol Sci. 2025 Jun 29;26(13):6287. doi: 10.3390/ijms26136287 (PMC12249835; doi:10.3390/ijms26136287)

## ALZHEIMER DISEASE

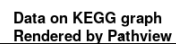

Data on KEGG graph  
Rendered by Pathview

Supplement: Supplementary file 1 [file ijms-26-06287-s001.zip › ijms-3639857-supplementary.pdf]
